# Retropharyngeal Calcific Tendinitis Mimicking a Retropharyngeal Phlegmon

**DOI:** 10.1155/2013/912628

**Published:** 2013-06-03

**Authors:** Nathalie Gabra, Manon Belair, Tareck Ayad

**Affiliations:** ^1^University of Montreal, Canada; ^2^Department of Radiology, Notre-Dame Hospital, University of Montreal Hospitals (CHUM), Montreal, QC, Canada H2L 4M1; ^3^Division of Otolaryngology—Head and Neck Surgery, Notre-Dame Hospital, University of Montreal Hospitals (CHUM), Montreal, QC, Canada H2L 4M1

## Abstract

*Background*. Acute retropharyngeal tendinitis is a little known but not an uncommon condition. It was first described by Hartley in 1964 as an inflammation of the longus colli muscle secondary to calcium crystals deposition on its insertion. The calcifications are mostly located on the oblique portion of the muscle at the level of C1-C2. *Methods*. We will describe this disease through 4 cases that presented in our institution. *Results*. The most common symptoms are severe neck pain, odynophagia, and a painful restriction of neck movement. It is associated with mild fever and inflammatory lab findings such as a slight elevation of white blood cell count, erythrocyte sedimentation rate, and C-reactive protein. CT scan is recommended as the first-line imaging modality to establish a diagnosis. Treatments consist of NSAIDs and analgesics to accelerate the healing process. If symptoms are severe, a course of corticosteroids is required. *Conclusion*. Since the clinical and laboratory findings of this condition and those of a retropharyngeal abscess overlap, it is important to establish the right diagnosis in order to prevent more invasive procedures. A good knowledge of this clinical entity by otolaryngologists would prevent delays in hospital discharge and unnecessary anxiety.

## 1. Introduction

Acute retropharyngeal tendinitis is a rare condition first described by Hartley in 1964 as an acute inflammation of the longus colli muscle secondary to calcium crystals deposition on the insertion of the muscle [1]. It is characterized by an acute onset of severe neck pain, odynophagia, and a painful restriction of neck movement. The presentation of this disease may be similar to more serious conditions such as retropharyngeal abscess, meningitis, cervical myopathy, and traumatic injury. Thus, knowledge of this condition by Otolaryngologists can prevent misdirected medical therapy, unnecessary invasive procedures, undue anxiety, and delays in hospital discharge. We describe herein 4 cases of retropharyngeal calcific tendinitis that presented in our institution. 

## 2. Case 1

A 63-year-old man presented with a 1-week history of sharp neck pain exacerbated by physical effort, odynophagia, and restricted neck movement in all directions. 

The flexible endoscopic examination revealed a swelling of the posterior wall of the nasopharynx with diffuse erythema. There was no pus or any suspicious lesions. Oropharynx, hypopharynx, and larynx were normal. His laboratory values included slight leukocytosis and an elevated C-reactive protein. 

**Figure 1:**
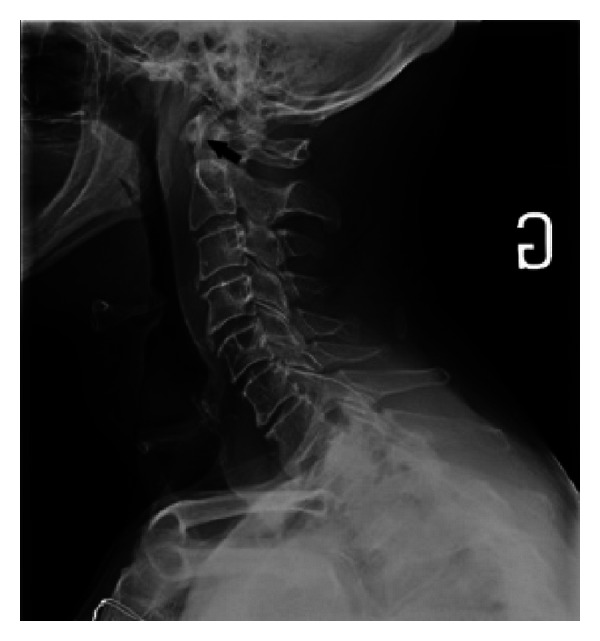
On the radiography of the neck, note the presence of an ill-defined calcification at the level of C1 (black arrow).

**Figure 2:**
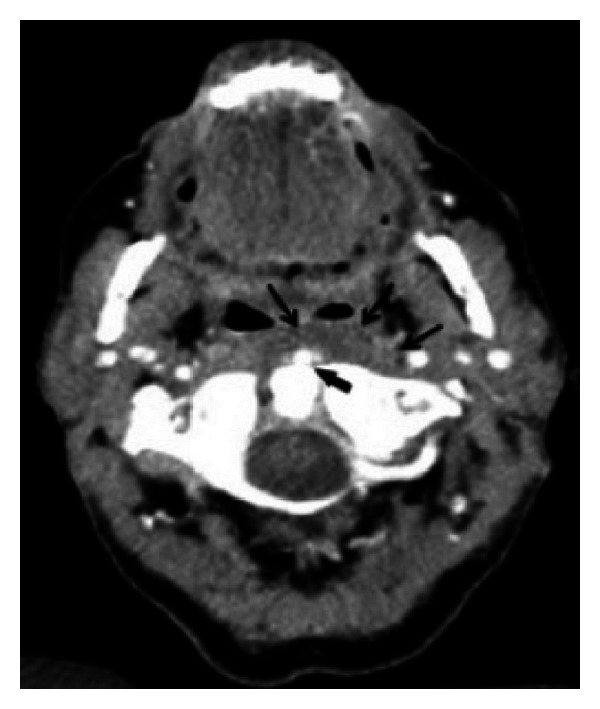
On the CT scan of the neck note the presence of an ill-defined calcification (thick black arrow) located in the left longus colli muscle at the level of C1. Note also the fat infiltration of the retropharyngeal space (thick black arrow).

On the same day, a cervical radiography and a contrast-enhanced CT scan were performed, and the radiologic differential diagnosis favored a retropharyngeal infectious process but a tumoral etiology was not excluded. A calcification at the superior insertion of the longus colli tendons at the C1 level was present on imaging but it was not initially identified (Figures 1 and 2). Therefore the patient was treated with a presumptive diagnosis of retropharyngeal phlegmon with IV steroids and piperacillin-tazobactam. He was discharged two days later with a 10-day course of oral antibiotics and a four-day course of prednisone. 

**Figure 3:**
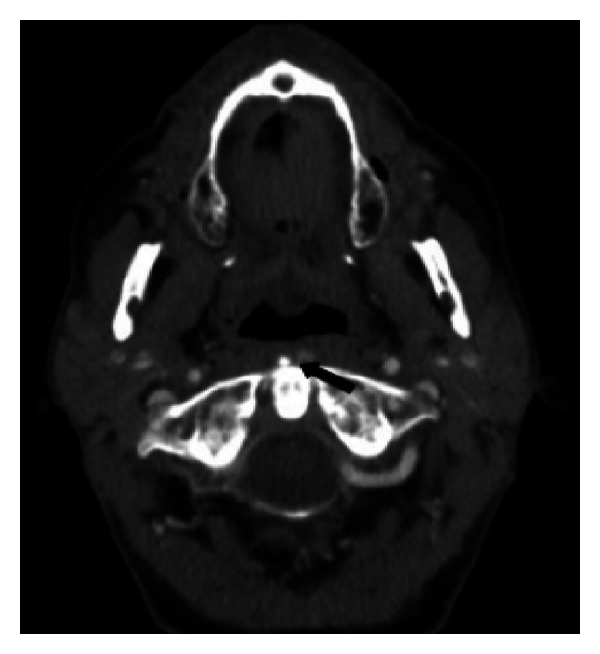
An important reduction of the swelling and the crystal deposit in the longus colli muscle after appropriate treatment with NSAIDs (black arrow).

 A control CT scan done 12 days later showed a net reduction of the retropharyngeal edema and also a significant reduction of the calcification (Figure 3). 

Twenty-five days later, the patient presented again with a mild left cervical pain and a slightly bulged posterior wall of the nasopharynx without erythema. Thus, it was suspected that the patient was initially misdiagnosed, and an MRI appeared within normal limits. At that time, the revision of the file by the neuroradiologist depicted the presence of the calcification and suggested the diagnosis of retropharyngeal calcific tendonitis. The patient was successfully treated with a course of oral NSAIDs and prednisone.

## 3. Case 2

 A 40-year-old women had a 4-day history of sharp neck pain (10/10) at the level of C3-C4 irradiating anteriorly and to the shoulder, limited neck movements, and odynophagia. She had a personal history of dyslipidemia and chronic lumbalgia and a family history of Sjören's syndrome, lupus, and fibromyalgia. She had no fever, and the ENT examination revealed a generalized oedema at palpation. The initial diagnosis was a neck torticollis, and the patient was discharged with a course of muscle relaxant and NSAIDs. 

 Two days later, she returned with laryngeal edema revealed on the flexible endoscopy examination and an increased neck pain. A contrast-enhanced CT scan revealed calcifications of the longus colli muscle at the C1-C2 level and inflammatory aspect of the retropharyngeal space without any fluid collection. The patient was then diagnosed with retropharyngeal calcific tendinitis and treated with a course of NSAIDs, diazepam, lorazepam, and analgesics (opioids) for 3 weeks and physiotherapy for 6 weeks (one session per week). Three weeks later, there were no symptoms, and the patient had completely recovered.

## 4. Case 3

A 36-year-old women had a 5-day history of neck pain exacerbated with movement, limited neck motion and odynophagia. At the ENT physical examination, there was a posterior cervical pain with palpation, and a swelling of the posterior pharyngeal wall was detected. 

 A cervical radiography was first performed, and a swelling of the retropharyngeal space was noted. The differential diagnosis favoured an upper respiratory tract infection or a retropharyngeal abscess. The patient received a course of dexamethasone and IV clindamycin with no further improvement. 

 A contrast-enhanced CT scan was done 4 days later, and calcifications on the longus colli muscle at C1-C2 level were detected. The patient was immediately diagnosed with retropharyngeal calcific tendinitis and treated with a course of NSAIDs and prednisone. Ten days later, the patient had completely recovered.

## 5. Case 4

A 52-year-old man had a 2-day history of neck pain, tenderness, odynophagia, limited range of motion, and sore throat. The patient has a history of pulmonary cystic fibrosis; he received lung transplantation and was taking corticosteroids. He had a normal count of white blood cells and a slightly elevated C-reactive protein.

A contrast-enhanced CT scan revealed a retropharyngeal space fluid expanding bilaterally and reaching the carotid space. A retropharyngeal phlegmon was suspected and the patient received with a 4-day course of IV piperacillin-tazobactam, IV vancomycin, and an increased dose of prednisone. 

 Two days later, the patient did not improve; therefore, it was suspected that he was initially misdiagnosed. Retrospective review of the CT scan revealed calcifications of the longus colli muscle. The patient was successfully treated with a 10-day course of prednisone and achieved a complete resolution of symptoms after treatment

The clinical and investigation findings of the four presented cases are summarized in Tables 1 and 2. 

## 6. Discussion

Retropharyngeal calcific tendonitis also called calcific prevertebral tendonitis or calcific tendonitis of the longus colli muscle is a little known but not an uncommon clinical syndrome [2]. A reactive inflammatory process induced by calcium hydroxyapatite deposition on the tendon of the longus colli muscle explains the clinical picture. Ring et al. in 1994 described 5 cases of retropharyngeal calcific tendinitis. A biopsy taken from one of these patients demonstrated a foreign-body inflammatory response to deposits of crystals of hydroxyapatite [3]. The calcifications are usually present in the superior oblique portion of the longus colli muscle at the C1-C2 levels [4] although a C5-C6 level has also been described [9].

This disease mostly affects adults between 30 and 60 years old although a range of 21 to 81 years were reported [5]. No race or ethnicity is overrepresented. The etiology of the disease is not fully understood, but it was reported that excessive mechanical pressure coupled with a pre-existing degenerative damage to the cervical spine, collagen vascular disorders, kidney failure, or osteoarthritis can result in an accumulation of crystals on the muscle.

According to the review of the literature done by Park et al., the most common symptoms were neck pain (94%), limited range of motion (45%), odynophagia (45%), neck stiffness (42%), dysphagia (27%), sore throat (17%), and neck spasm (11%). Laboratory findings include a slightly elevated white blood cells count, erythrocyte sedimentation rate, and C-reactive protein [6, 7]. 

Endoscopic physical examination often shows swelling of the posterior wall of the nasopharynx. Imaging studies reveal edema of the prevertebral space sometimes associated with a collection of fluid expanding in the retropharyngeal space. Since many infectious or tumoral processes can involve the prevertebral space, these findings do not help us through the differential diagnosis. In fact, the clinical and laboratory findings of retropharyngeal calcific tendonitis and those of an acute infection of the retropharyngeal space may overlap [8].

 Reviewing the findings on imaging studies can help differentiate between both entities. In retropharyngeal tendonitis there is an absence of suppurative lymph nodes with low-attenuation centres as it is seen in a retropharyngeal abscess [9]. The key observation is the presence of calcifications on the tendon of the longus colli muscle at the C1-C2 level, which definitely confirms the diagnosis [9]. CT scan is more sensitive than MRI and radiographs in revealing the prevertebral calcifications [7]. CT is also more accessible and less expensive than MRI; it is thus recommended as first-line imaging modality. 

 Retropharyngeal calcific tendonitis is a benign, self-limiting disease that rarely requires admission, whereas in retropharyngeal abscess is associated with a high morbidity, antibiotics, and surgical drainage might be required. 

 In conclusion, retropharyngeal calcific tendonitis is a rare benign condition that needs to be recognized by otolaryngologists in order to prevent anxiety, misdirected therapy and unnecessary invasive procedures. The presence of a nonspecific fluid collection and an inflammatory aspect of the retropharyngeal space require a review of the radiographic studies to determine the presence of calcification on the longus colli muscle. Despite the fact that it is a benign and self-limiting disorder, it can cause airway deficiency and can be very painful for the patient if it remains untreated [10]. Recommended treatments consist of NSAIDs and supportive care to accelerate the healing process. If symptoms are severe, a course of corticosteroids is required [8]. Calcium deposit should resolve in a week or two [11], and the patients become symptom-free after 1–3 weeks [12]. Further studies should be done to elucidate the etiology and pathogenesis of this disease.

## Figures and Tables

**Table 1:** Summary of clinical findings.

Clinical finding	Case *1*	Case *2*	Case *3*	Case *4*	Ratio and mean
Age (years)	63	40	30	52	46
Sex	M	F	F	M	1 : 1
RCT immediately identified	−	−	−	−	0/4
Time between presentation and diagnosis (days)	25	2	4	2	9.25
Neck pain	+	+	+	+	4/4
Limited ROM	+	+	+	+	4/4
Odynophagia	+	+	+	+	4/4
Dysphagia	−	−	−	−	0/4
Severe fever (≥38°C)	−	−	−	−	0
Leukocytosis (≥10 800 mm^3^)	+	N/A	N/A	−	1/2

RCT: retropharyngeal calcific tendinitis.

ROM: range of movements.

**Table 2:** Flexible endoscopy, cervical radiography and CT scan findings summary anterior to C1–C4 in the prevertebral space.

Flexible endoscopy and CT scan findings	Case *1*	Case *2*	Case *3*	Case *4*	Ratio
Soft tissue inflammation (swelling and erythema)	+	+	+	+	3/3
Calcification	+	+	+	+	4/4
Effusion or fluid collection	−	−	−	+	1/4
